# Progress Toward Prevention of Transfusion-Transmitted Hepatitis B and Hepatitis C Infection — Sub-Saharan Africa, 2000–2011

**Published:** 2014-07-25

**Authors:** Ibironke W. Apata, Francisco Averhoff, John Pitman, Adam Bjork, Junping Yu, Noryati Abu Amin, Neelam Dhingra, Amy Kolwaite, Anthony Marfin

**Affiliations:** 1 EIS officer, CDC; 2 Division of Global HIV/AIDS, Center for Global Health, CDC; 3 Division of Viral Hepatitis, National Center for HIV/AIDS, Viral Hepatitis, STD, and TB Prevention, CDC; 4 Blood and Transfusion Safety, World Health Organization

Infections with hepatitis B virus (HBV) and hepatitis C virus (HCV) are major causes of morbidity and mortality globally, primarily because of sequelae of chronic liver disease including cirrhosis and hepatocellular carcinoma (1). The risks for HBV and HCV transmission via blood transfusions have been described previously (2) and are believed to be higher in countries in sub-Saharan Africa (3). Reducing the risk for transfusion-transmitted human immunodeficiency virus (HIV), HBV, and HCV infection is a priority for international aid organizations, such as the U.S. President’s Emergency Plan for AIDS Relief (PEPFAR), the Global Fund to Combat HIV/AIDS, Malaria, and Tuberculosis, and the World Health Organization (WHO). Over the last decade, PEPFAR and the Global Fund have supported blood safety programs in many sub-Saharan African countries with heavy burdens of HIV and acquired immunodeficiency syndrome (AIDS), hepatitis, malaria, and maternal mortality. This report summarizes HBV- and HCV-related surveillance data reported by the blood transfusion services of WHO member states to WHO’s Global Database on Blood Safety (GDBS) (4). It also evaluates the performance of blood safety programs in screening for HBV and HCV in 38 sub-Saharan Africa countries.* Selected GDBS indicators were compared for the years 2000 and 2004 (referred to as the 2000/2004 period) and 2010 and 2011 (referred to as the 2010/2011 period). From 2000/2004 to 2010/2011, the median of the annual number of units donated per country increased, the number of countries screening at least 95% of blood donations for HBV and HCV increased, and the median of the national prevalence of HBV and HCV marker-reactive blood donations decreased. These findings suggest that during the past decade, more blood has been donated and screened for HBV and HCV, resulting in a safer blood supply. Investments in blood safety should be continued to further increase the availability and safety of blood products in sub-Saharan Africa.

Since 1998, WHO member states have submitted blood safety and availability indicators to GDBS. The database contains 49 variables related to blood donations, including screening for HBV, HCV, HIV, and syphilis. Data are self-reported from each country’s routine blood collection and testing operations, which typically are conducted at blood transfusion service facilities and then sent to WHO, usually on an annual or biennial basis. At the time of this analysis, GDBS contained data for the following years: 2000, 2004, 2006, 2008, 2010, and 2011.

The years 2000/2004 and 2010/2011 were selected for analysis because these periods correspond to the earliest and latest GDBS data available at the time of analysis. Data available for 38 sub-Saharan African countries were analyzed, including the median number of blood donations per year for 2000/2004 and 2010/2011, the number of donations screened for hepatitis B surface antigen (HBsAg) and hepatitis C antibody (anti-HCV), and the number and proportion of donations that were reported as HBsAg-reactive and anti-HCV-reactive. For the purpose of this analysis, the term marker-reactive (i.e., HBsAg-reactive or anti-HCV reactive) was used because data on confirmatory test results were not collected. Country-specific means were calculated for the percentage of blood donations screened for HBV and HCV during the 2000/2004 and 2010/2011 periods. Screening percentages for both HBsAg and anti-HCV were classified into one of three categories: 95%–100%, 80%–94%, or <80%. The prevalence of HBV and HCV marker-reactive donations was calculated as the total number of marker-reactive donations for each period (2000/2004 or 2010/2011) divided by the total number of donations for each period. To quantify changes in the prevalence of marker-reactive donations from 2000/2004 to 2010/2011, rate ratios (2010/2011 to 2000/2004) of HBV and HCV infection prevalence were calculated for each country. The z-test was used to determine if the rate ratio reflected a statistically significant change (p<0.05). If data were missing for either 2000 or 2004, the data for the single available year were used in the analysis. Similarly, if data were missing for 2010 or 2011, the data for the single available year were used. If data were missing for both years in either period, the country was excluded from the analysis. For this reason, the numbers of countries in the comparisons across periods were not always the same for all variables analyzed.

The median number of donations increased from 31,368 units (36 countries; interquartile range [IQR] = 12,987–80,629 units) to 86,328 units (38 countries; IQR = 30,139–139,207) from 2000/2004 to 2010/2011, and the number of countries testing at least 95% of donations for HBsAg increased from 29 (76%) of 38 countries to 33 (94%) of 35 countries during the same interval. The number of countries testing at least 95% of donations for HCV antibody increased from 13 (34%) of 38 reporting countries to 30 (86%) of 35 countries from 2000/2004 to 2010/2011.

The median percentage of HBV marker-reactive blood donations was 7.1% (36 reporting countries; IQR = 4.1%–11.1%) in 2000/2004 and 4.4% (36 countries; IQR = 2.2%–7.4%) in 2010/2011 (Figure 1, Table 1). The median percentage of anti-HCV marker-reactive donations was 1.4% (31 countries; IQR = 0.6%–3.1%) and 0.9% (36 countries; IQR = 0.5%–1.7%) in 2000/2004 and 2010/2011, respectively (Figure 2, Table 2). From 2000/2004 to 2010/2011, 28 (82%) of 34 reporting countries reported a statistically significant (p<0.05) decrease in HBsAg marker-reactive donations, and 14 (48%) of 29 reporting countries reported a significant decrease in anti-HCV marker-reactive donations. Overall, combined data from all countries showed a 37% decrease (p=0.07; 34 reporting countries) in the proportion of HBsAg-reactive donations and a 51% decrease (p=0.04; 29 reporting countries) in the proportion of anti-HCV-reactive donations between the periods analyzed.

## Discussion

This report highlights substantial increases in the number of blood units donated in sub-Saharan Africa, a region known to have blood shortages (5). It also describes increases in the number of countries testing for HBsAg and HCV antibody, and decreases in the proportion of donations screening positive for markers of HBV and HCV, likely reducing the risk for HBV and HCV infection through blood transfusions in sub-Saharan Africa during the last decade. To reduce the risk for transfusion-transmitted infection and increase the availability of blood, WHO recommends implementation of an integrated and comprehensive strategy based on five key elements: 1) establish a nationally coordinated blood transfusion service, 2) collect blood from regular, voluntary, non-remunerated donors from low-risk populations, 3) test for transfusion-transmissible infections, blood group, and compatibility using quality-assured procedures, 4) reduce unnecessary transfusion through appropriate use of blood, and 5) implement quality systems for the entire transfusion process, from donor recruitment to the follow-up of the recipients of transfusion (5,6). PEPFAR-support for blood transfusion service programs based on WHO recommendations have been shown to reduce the risk for HIV transmission via transfusion while increasing the supply of safe blood (5). However, not all countries are reporting screening at least 95% of their blood donations for HBV and HCV, and high rates of HBV and HCV infection among donors were noted in some countries, indicating continued risk for transfusion recipients. Two previous reports have shown an increase in HIV screening, an increase in donations, and a decrease in the prevalence of HIV-positive donations in African countries (5,7). This report demonstrates that many African countries have made similar progress with screening donations for HBV and HCV and decreasing the prevalence of HBV and HCV marker-reactive donations.

The epidemiology of HBV and HCV infection is poorly described in sub-Saharan Africa. The findings in this report offer additional data to better understand the burden of HBV and HCV infection in the region. Marker-reactive rates of HBV among blood donors were high, with most countries having rates exceeding 3%; countries of West Africa had particularly high rates, several with rates exceeding 10%. Rates for HCV infection were generally lower, most with rates less than 2%. The risk for developing chronic HBV infection is greatest when infection occurs during birth (up to 90%) and during childhood (30%), and most chronic HBV infection in sub-Saharan Africa is thought to be the result of transmission during birth or childhood (9). Chronic hepatitis C develops in up to 85% of those who are infected with HCV (9). Coinfection with HIV increases the risk for HBV- and HCV-related liver disease. The risk factors for transmission of HBV and HCV infection in sub-Saharan Africa might include receipt of medical or dental care associated with poor infection control practices, injection drug use, receipt of contaminated blood products, and scarification. Childbirth, inapparent exposures during childhood, and sexual exposure pose a greater risk for HBV than HCV. Because risk factors for transmission of HBV and HCV in sub-Saharan Africa have not been well described, screening by blood collection agencies for recognized risk behaviors, such as injection drug use, might not be as helpful in identifying most cases of chronic HBV or HCV infection in sub-Saharan Africa compared with other parts of the world.

The findings in this report are subject to at least five limitations. First, the data are self-reported by each country and cannot be independently verified. Second, the quality of laboratory screening is not known and might vary within and between countries and between the 2000/2004 and 2010/2011 periods. However, in some PEPFAR countries, efforts to improve the quality of laboratory screening for transfusion-transmissible infections, such as proficiency testing, have been implemented. Third, the data do not represent all health facilities (e.g., private, faith-based, or military facilities) that collect blood outside the national blood transfusion service network. Fourth, countries that had missing data for both years in 2000/2004 or 2010/2011 (four countries for HBV and nine for HCV) were excluded from the comparison of the overall changes in prevalence of HBsAg and anti-HCV reactive blood donations in sub-Saharan Africa. Finally, the screening does not include testing persons for evidence of active HCV infection (i.e., HCV RNA).

What is already known on this topic?

In sub-Saharan Africa and other resource-limited settings, transfusion-transmitted hepatitis B virus (HBV) and hepatitis C virus (HCV) infections remain a public health burden. Reducing the prevalence of hepatitis virus infections in donated blood is a priority for countries seeking ways to increase the safety and adequacy of national blood supplies.

What is added by this report?

From 2000 to 2011, the number of countries in sub-Saharan Africa screening at least 95% of donated blood units for HBV and HCV increased from 76% to 94% and 34% to 86%, respectively. During the same period, the median percentage of HBV marker-reactive units decreased from 7.1% to 4.4%, and the median percentage of HCV marker-reactive units decreased from 1.4% to 0.9%.

What are the implications for public health practice?

This study provides important data and highlights trends to help focus existing and future strategies and investments by national governments and global health programs to reach countries’ goals for safe and adequate blood supplies. The analyses demonstrate the continued risk for transfusion-transmitted HBV and HCV infections throughout sub-Saharan Africa. Although great progress in reducing this risk has been made in some of these countries, substantial progress in others is yet to be seen.

Improving the quality of laboratory screening of blood for HBV and HCV is only one component in reducing the risk for transfusion-transmitted HBV and HCV. Critical adjuncts to laboratory screening for improved blood safety include 1) targeting outreach and blood collection efforts among populations with low-risk behavioral profiles, 2) collecting blood from regular, voluntary, non-remunerated donors, 3) providing educational materials in donation settings to help infected persons defer themselves from donation (self-deferral) without publicly disclosing their infection status, 4) providing post-donation counselling and referral to care and treatment for blood donors who screen positive for transfusion-transmissible infections, and 5) increasing the proper use of donor history questionnaires to defer persons with high-risk behaviors. Monitoring the prevalence of transfusion-transmissible infections among blood donors is one way to measure the effectiveness of these risk-reduction strategies. Data showing significantly reduced prevalence of laboratory-detected transfusion-transmissible infections suggests improvements in donor recruitment and selection practices.

During the 2010/2011 period, six countries reported high percentages (i.e., 10%–19%) of blood donations to be marker-reactive for HBV, and one country reported a high percentage (i.e., exceeding 5%) of its blood donations to be marker-reactive for HCV. Rates of HBV and HCV marker-reactive donations indicate that regional prevalence of chronic HBV and HCV infections remain high among blood donors. Although surveillance of infectious disease rates among blood donors might be of benefit to blood services and public health agencies, reductions in prevalence among blood donors might not be indicative of similar changes among the general population. Despite the progress described in this report, sustained commitment to blood safety programs will be required to further decrease the risk for transfusion-transmitted infections throughout sub-Saharan Africa.

## Figures and Tables

**FIGURE 1:**
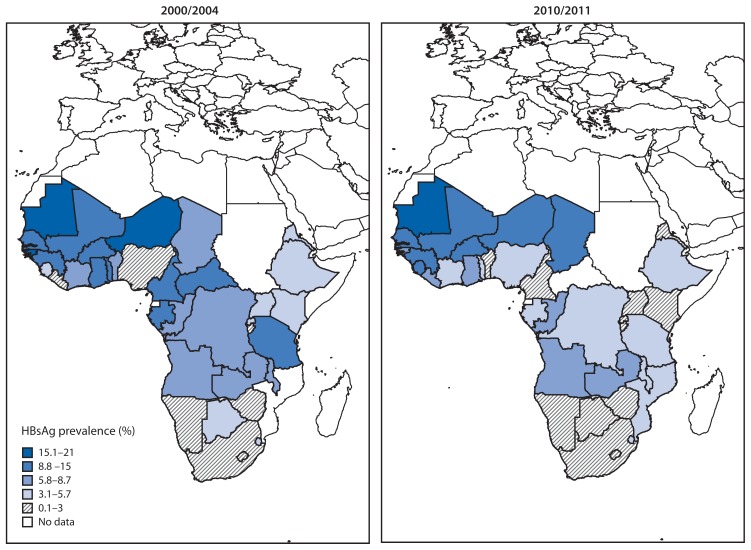
Prevalence of HBsAg-reactive blood donations, by country — sub-Saharan Africa, 2000/2004 and 2010/2011 **Abbreviation:** HBsAg = hepatitis B surface antigen. **Source:** Global Database for Blood Safety.

**FIGURE 2:**
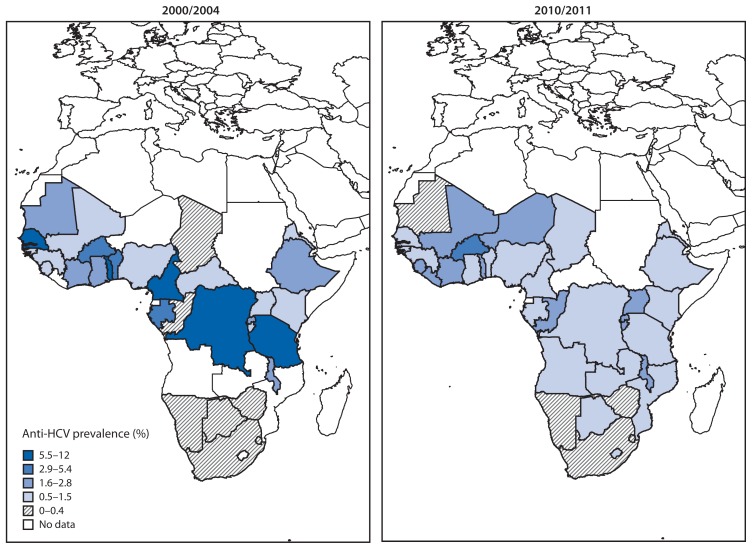
Prevalence of anti-HCV reactive blood donations, by country — sub-Saharan Africa, 2000/2004 and 2010/2011 **Abbreviation:** anti-HCV = hepatitis C antibody. **Source:** Global Database for Blood Safety.

**TABLE 1:** HBV prevalence in blood donations (i.e., donations reactive for HBsAg), by country — sub-Saharan Africa, 2000/2004 and 2010/2011

Country	2000/2004		2010/2011		Ratio of HBV prevalence (2010/2011: 2000/2004)*	Direction of change
	
	HBV prevalence (%)	Total donations	HBV prevalence (%)	Total donations		
Angola	8.68	78,000	6.74	78,275	0.78	↓
Benin	7.51	62,949	1.65	122,675	0.22	↓
Botswana	4.21	25,210	2.21	36,930	0.52	↓
Burkina Faso	12.48	64,620	9.85	140,706	0.79	↓
Burundi	2.79	N/A	2.77	76,301	N/A	N/A
Cameroon	15.00	70,000	1.34	54,248	0.09	↓
Central African Republic	10.45	10,600	N/A	14,500	N/A	N/A
Chad	7.76	5,000	10.10	30,123	1.30	↑
Republic of the Congo	6.40	31,756	7.35	94,020	1.15	↑
Côte d’Ivoire	6.93	139,031	5.31	194,775	0.77	↓
Democratic Republic of the Congo	7.31	21,740	3.43	722,577	0.47	↓
Eritrea	3.60	12,500	2.27	20,686	0.63	↓
Ethiopia	4.00	24,000	3.42	94,218	0.86	↓
Gabon	10.49	25,500	4.57	30,186	0.44	↓
Gambia	N/A	12,153	N/A	17,880	N/A	N/A
Ghana	11.75	130,000	6.58	194,399	0.56	↓
Guinea	11.20	23,430	9.79	53,110	0.84	↓
Guinea-Bissau	18.42	3,601	6.1	2,970	0.33	↓
Kenya	5.31	210,000	1.75	244,228	0.33	↓
Lesotho	1.37	6,600	0.90	9,675	0.66	↓
Liberia	0.50	N/A	7.40	13,472	N/A	N/A
Malawi	6.90	24,000	3.43	122,132	0.50	↓
Mali	11.33	45,000	14.27	94,819	1.26	↑
Mauritania	21.00	3,846	18.82	17,259	0.90	↓
Mozambique	N/A	114,223	5.30	222,087	N/A	N/A
Namibia	2.41	37,235	0.78	23,338	0.32	↓
Niger	20.00	7,000	11.78	103,238	0.59	↓
Nigeria	3.00	60,000	4.12	93,863	1.37	↑
Rwanda	4.39	55,433	1.75	78,793	0.40	↓
Senegal	10.50	44,400	10.51	105,816	1.00	↑
Sierra Leone	5.73	13,149	11.60	29,114	2.02	↑
South Africa	0.28	1,700,000	0.12	1,872,095	0.42	↓
Swaziland	4.81	16,500	3.11	21,328	0.65	↓
Togo	11.48	18,884	3.46	73,195	0.30	↓
Uganda	5.00	110,000	2.28	383,985	0.46	↓
Tanzania	11.00	8,437	5.11	189,740	0.47	↓
Zambia	7.56	88,514	6.02	168,295	0.80	↓
Zimbabwe	1.56	150,000	0.92	134,709	0.59	↓
*Median*	*7.12*	*—*	*4.35*	*—*	*—*	

**Abbreviations:** HBV = hepatitis B virus; HBsAg = hepatitis B surface antigen; N/A = not available (missing or incomplete data).

**Source:** Global Database for Blood Safety.

* 2010/2011:2000/2004 prevalence ratios are statistically significant at p<0.05 for all countries except Senegal.

**TABLE 2:** HCV prevalence in blood donations (i.e., donations reactive for anti-HCV), by country — sub-Saharan Africa, 2000/2004 and 2010/2011

Country	2000/2004		2010/2011		Ratio of HCV prevalence (2010/2011: 2000/2004)*	Direction of change
	
	HCV prevalence (%)	Total donations	HCV prevalence (%)	Total donations		
Angola	N/A	78,000	0.57	78,275	N/A	N/A
Benin	3.82	62,949	0.53	122,675	0.14	↓
Botswana	0.34	25,210	0.49	36,930	1.41	↑
Burkina Faso	4.58	64,620	5.21	140,706	1.14	↑
Burundi	1.41	N/A	1.54	76,301	N/A	N/A
Cameroon	10.00	70,000	0.76	54,248	0.08	↓
Central African Republic	1.20	7,000	N/A	14,500	N/A	N/A
Chad	0.20	3,000	0.51	30,123	2.56	↑
Republic of the Congo	0.40	31,756	1.98	94,020	4.92	↑
Côte d’Ivoire	2.29	139,031	1.56	194,775	0.68	↓
Democratic Republic of the Congo	7.20	17,138	1.46	722,577	0.21	↓
Eritrea	0.88	12,500	0.53	20,686	0.60	↓
Ethiopia	2.00	24,000	0.47	94,218	0.23	↓
Gabon	5.39	25,500	0.77	30,186	0.14	↓
Gambia	N/A	12,153	N/A	17,880	N/A	N/A
Ghana	2.40	70,000	1.00	194,399	0.42	↓
Guinea	0.60	11,430	1.07	53,110	1.78	↑
Guinea-Bissau	0.70	1,739	0.80	2,970	1.08	↑
Kenya	0.70	120,000	0.78	244,228	1.12	↑
Lesotho	N/A	6,600	0.81	9,675	N/A	N/A
Liberia	N/A	N/A	2.30	13,472	N/A	N/A
Malawi	2.00	24,000	2.00	122,132	1.00	↑
Mali	1.00	45,000	2.20	94,819	2.20	↑
Mauritania	1.78	7,855	0.02	9,164	0.01	↓
Mozambique	N/A	114,223	0.91	222,087	N/A	N/A
Namibia	0.03	37,235	0.09	22,018	2.60	↑
Niger	N/A	7,000	2.02	103,238	N/A	N/A
Nigeria	1.50	60,000	1.31	93,863	0.88	↓
Rwanda	2.83	55,433	1.97	78,793	0.70	↓
Senegal	12.00	19,400	0.63	105,816	0.05	↓
Sierra Leone	0.67	13,149	2.20	29,114	3.25	↑
South Africa	0.04	1,700,000	0.01	1,872,095	0.14	↓
Swaziland	0.01	16,500	0.25	21,328	14.18	↑
Togo	8.04	18,884	1.83	73,195	0.23	↓
Uganda	0.75	110,000	1.71	383,985	2.28	↑
Tanzania	8.00	8,437	0.55	189,740	0.07	↓
Zambia	N/A	88,514	0.93	168,295	N/A	N/A
Zimbabwe	0.03	80,000	0.34	134,709	11.41	↑
*Median*	*1.41*	*—*	*0.86*	*—*	*—*	*—*

**Abbreviations:** HCV = hepatitis C virus; anti-HCV = hepatitis C antibody; N/A = not available (missing or incomplete data).

**Source:** Global Database for Blood Safety.

* 2010/2011:2000/2004 prevalence ratios are statistically significant at p<0.05 for all countries except Guinea-Bissau.

## Footnotes

* Angola, Benin, Botswana, Burkina Faso, Burundi, Cameroon, Central African Republic, Chad, Republic of the Congo, Côte d’Ivoire, Democratic Republic of the Congo, Eritrea, Ethiopia, Gabon, Gambia, Ghana, Guinea, Guinea-Bissau, Kenya, Lesotho, Liberia, Malawi, Mali, Mauritania, Mozambique, Namibia, Niger, Nigeria, Rwanda, Senegal, Sierra Leone, South Africa, Swaziland, Tanzania, Togo, Uganda, Zambia, and Zimbabwe.
